# No evidence for age-related differences in mitochondrial RNA quality in the female germline

**DOI:** 10.1530/RAF-22-0025

**Published:** 2022-08-24

**Authors:** Fiona Hartley, Arwa Alageel, Ruth Appeltant, Nicki Gray, Emmanouela Repapi, Dagan Wells, Suzannah A Williams, Joanna Poulton

**Affiliations:** 1 Department of Oncology, University of Oxford, Oxford, UK; 2 Nuffield Department of Women’s and Reproductive Health, University of Oxford, Oxford, UK; 3 Juno Genetics, Winchester House, Oxford, UK; 4 Analysis, Visualisation & Informatics Group / Medical Research Council Molecular Haematology Unit at the University of Oxford MRC Weatherall Institute of Molecular Medicine, John Radcliffe Hospital, Headington, Oxford OX3 9DS

**Keywords:** mitochondrial RNA, heteroplasmy, oocytes, cumulus cells, RNA-seq, RNA modification

## Abstract

**Graphical abstract:**

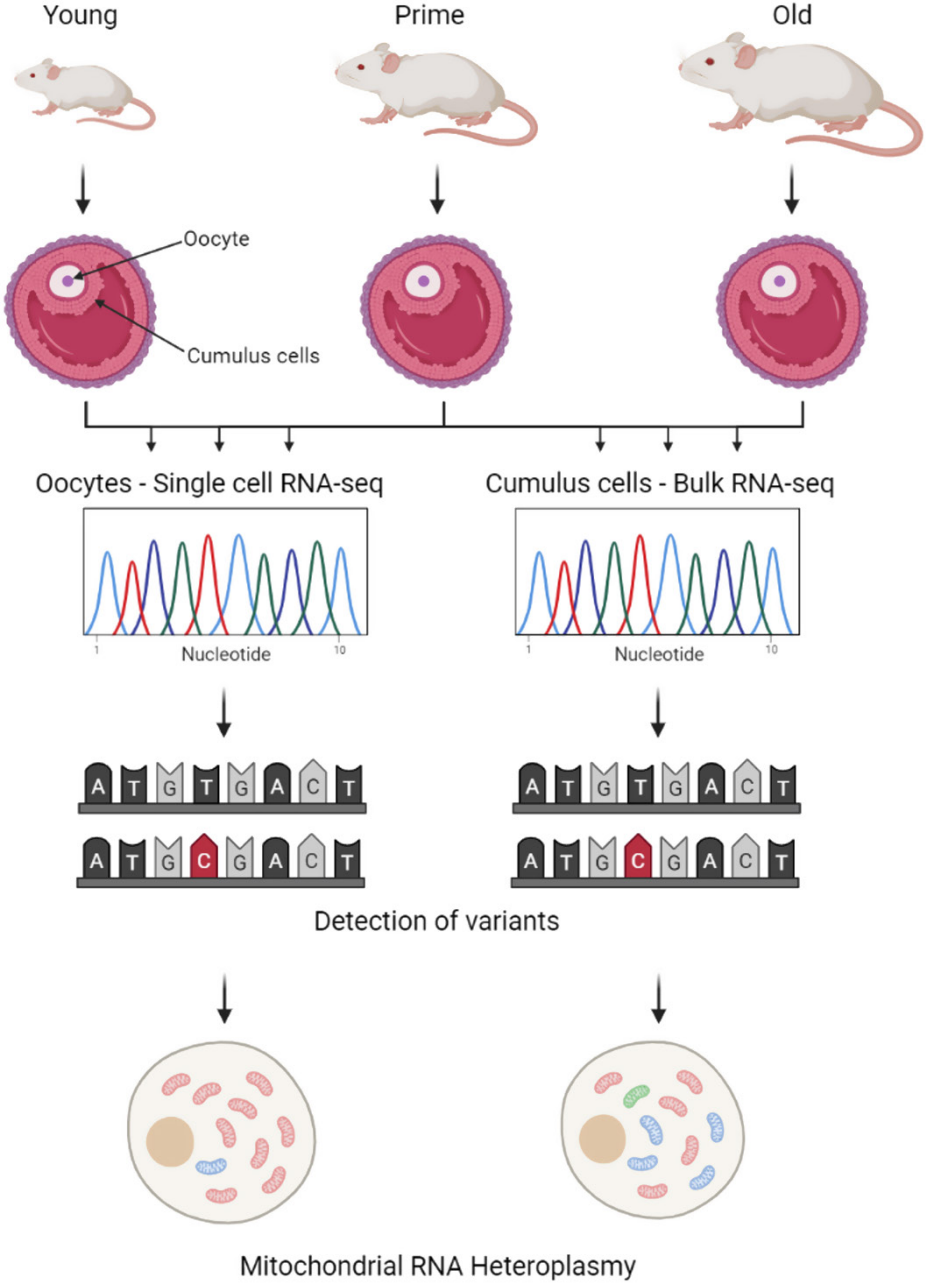

**Abstract:**

Mitochondrial quality is implicated as a contributor to declining fertility with aging. We investigated mitochondrial transcripts in oocytes and their associated cumulus cells from mice of different ages using RNA-seq. Mice aged 3 weeks, 9 weeks, and 1 year were superovulated, and 48 h later, oocyte cumulus complexes were collected by follicle puncture. We did not detect any major differences that could be attributed to aging. However, mitochondrial RNA transcripts which deviated from the consensus sequence were found at a higher frequency in cumulus cells than in their corresponding oocyte. Previous investigations have shown that variation in the sequence of mtRNA transcripts is substantial, and at least some of this can be accounted for by post-transcriptional modifications which impact base calling during sequencing. Our data would be consistent with either less post-transcriptional modification in mitochondrial RNA from oocytes than cumulus cells or with lower mtDNA mutational load.

**Lay summary:**

Women become less fertile as they age. Shortage of energy contributes to this, caused by a decline in the quality of mitochondria (the powerhouses of the cell) in the egg. Genes are the blueprint for the cell. They are made of DNA which is copied into an RNA message, or instructions, for making proteins. We counted differences in the RNA message of developing eggs and the cells that support them during development (cumulus cells). We compared the number of these differences in mice of different ages. These age groups represent mice had not reached puberty, those of prime reproductive age, and old mothers. We did not find any differences linked to the age of the mice. However, we did find differences between the egg and the cumulus cells. In most cases, there were lower levels of mutations in eggs than there were in cumulus cells.

## Introduction

Women’s fertility declines with aging. Maintaining mitochondrial quality in oocytes is important in human fertility, as well as in rare disorders arising from maternally inherited mitochondrial DNA (mtDNA) mutations. While aneuploidy and declining oocyte numbers explain a substantial component of the decline, there is likely to be an important mitochondrial component. This is because fertility is impaired in mice that transmit a high load of mitochondria with damaged mtDNA to the embryo (Ross *et al.* 2013). Homoplasmy, where all mtDNAs are identical, was initially held to be the norm in healthy individuals, but-next generation sequencing has clearly revealed a low level of mtDNA heteroplasmy (more than one type of mtDNA co-existing in the same individual) in normal controls. Indeed, low levels of heteroplasmy that are commonly detectable in post-mitotic tissues have been implicated in aging (Cortopassi & Arnheim 1990). For instance, the mtDNA mutations that accumulate in post-mitotic tissues such as muscle and brain may contribute to declining function. Evidence is now emerging that mutations could accumulate in a similar manner in oocytes over time (Yang *et al.* 2020). Moreover, there are many gaps in our knowledge of how mitochondrial quality is maintained (Cox *et al.* 2021, Bhardwaj *et al.* 2022).

We were the first to demonstrate clonally expanded mtDNA in human and mouse oocytes, following a mitochondrial bottleneck during oogenesis/oocyte development (Marchington *et al.* 1997). While there is good indirect evidence for mtDNA quality control in the human (Li *et al.* 2016, Wei *et al.* 2019, Zaidi *et al.* 2019) and mouse (Ma *et al.* 2018) female germline, and direct evidence in mouse studies (Arbeithuber *et al.* 2020, Yang *et al.* 2020), little is known about the extent of sequence variation in the mitochondrial transcriptome. Studies that have used ultra-deep sequencing of mitochondrial RNA (mtRNA) have identified remarkable levels of sequence variation within individuals, as well as sites that show consistent patterns of post-transcriptional modification (Hodgkinson *et al.* 2014, Bar-Yaacov *et al.* 2016). Expression of nuclear-encoded genes potentially drives such modifications (Hodgkinson *et al.* 2014, Silzer *et al.* 2020). To our knowledge, there are no published RNA-seq data on oocyte mitochondrial transcripts.

Like humans, mice also exhibit an age-related decline in oocyte quality and fertility. C57BL/6 are a well-characterised inbred strain of mouse, in which reproductive potential declines with age. We analysed the variants in mitochondrial transcripts of oocytes and their corresponding cumulus cells as part of a larger study investigating gene expression in mouse oocytes. Variants found in the sequence of mtRNA can be caused by mutations in mtDNA or caused by post-transcriptional modification of the RNA, which leads to sequencing artefacts (Hodgkinson *et al.* 2014, Bar-Yaacov *et al.* 2016, Schwartz & Motorin 2017). In the present study, we refer to variation in mtDNA sequence as allele frequency or mtDNA heteroplasmy and in variation at the RNA level as mtRNA variants or mtRNA heteroplasmy. We refer to level of mtRNA heteroplasmy as ‘quality’ because this term can include variants resulting either from post transcriptional modification or to mtDNA heteroplasmy.

## Methods

We analysed mitochondrial transcripts as part of a larger study investigating gene expression in mouse oocytes (in preparation). The data for this study have been deposited in the European Nucleotide Archive (ENA) at EMBL-EBI under accession number PRJEB55346 (https://www.ebi.ac.uk/ena/browser/view/PRJEB55346).

### Ethics

This study was carried out with approval by the Local Ethical Review Panel (University of Oxford, Establishment license 30/2306) in accordance with the UK Animals (Scientific Procedures) Act 1986 within project licences granted by the Home Office and held by Prof Williams (project licence number 30/3352).

### Animals

Female C57BL/6 mice were purchased from Charles River UK Ltd and used at 3 weeks (prepubertal; young) and 9 weeks of age (prime reproductive age). Female C57BL/6 mice were also purchased at 10 months of age (Envigo, Netherlands) and maintained to 12 months of age (old). Mice were superovulated by i.p. administration of 5 IU pregnant mare serum gonadotrophin (Invitrogen) 48 h prior to ovary collection. Mice were killed by cervical dislocation and ovaries with associated bursa and fallopian tubes were isolated and individually placed into Dulbecco’s Buffered Saline free of calcium and magnesium (PBS) (Gibco, UK) and taken to the laboratory on ice for processing (less than 15 min).

### Oocyte and cumulus cell isolation

Ovaries were dissected from the bursa in PBS and washed. Large follicles were identified and oocyte-cumulus complexes (OCC) were obtained by puncturing follicles with a 30G needle. OCC’s from each mouse were placed into separate dishes in a droplet of PBS/BSA. Each OCC was transferred through a series of 10 µl PBS/BSA droplets to ensure removal of any loosely attached follicular cells. OCCs with tight layers of cumulus cells were selected for analysis.

Each OCC was processed separately. The cumulus cells were stripped from the oocyte by pipetting through a heat-stretched glass microcapillary pipette (Hirschmann® microcapillary pipette, Sigma-Aldrich, UK) and isolated as described below. Each denuded oocyte was washed through a series of PBS/BSA droplets. The zona pellucida was removed by a brief (<30 s) incubation in acid tyrodes solution (Cat. No. T1788, Sigma-Aldrich, UK) followed by washing through three sequential droplets of PBS/BSA. Each denuded zona-free oocyte was added individually to a microcentrifuge tube containing 4.45 µl lysis buffer according to the Smart-Seq2 protocol (Picelli *et al.* 2014). After preparing the lysis buffer, it was kept frozen until sample processing began. The lysis buffer was stored in fresh ice until sample loading. The microcentrifuge tube was then centrifuged for 30 s at 2000g to make sure the oocyte was immersed in the lysis buffer. The lysed oocyte samples were then placed on dry ice and subsequently stored at -80 ˚C.

The cumulus cells stripped from each oocyte (as described above) were collected in PBS/BSA and placed in a microcentrifuge tube. These were centrifuged at 120g for 3 min and the supernatant discarded. Samples of 0.5 µl of cells were removed from the cell pellet and added to a microcentrifuge tube containing 4.45 µl lysis buffer.

### Reverse transcription and PCR amplification

In order to convert the lysate into single-stranded cDNA, the lysate was added to a reaction mixture of SuperScript II reverse transcriptase, RNAse inhibitor, SuperScript II first-strand buffer, DTT, betaine, MgCl2, TSO and nuclease-free water. The samples were subsequently incubated at 42°C for 90 min, 10 repeated cycles at 50°C for 2 min, at 42°C for 2 min and finally at 70°C for 15 min. RT was followed by template switching and PCR preamplification. The reaction mixture consisted of KAPA HIFI HS Ready Mix, IS PCR primers and water. The protocol applied 3 min at 98°C, 15 s at 67°C and 15 min at 72°C. The PCR products were cleaned using Ampure SPRI beads (Beackman Coulter, cat. no. A63881). After incubation with beads, the supernatant was removed, ethanol was briefly added twice and the samples were dried for 8 min. Supernatants were collected after incubation in an elution buffer.

### Analysis of integrity – Qubit, Bioanalyser, PicoGreen and TapeStation

The amplified cDNA of oocytes and cumulus cells was subjected to quality and quantity analysis using the Qubit® 2.0 fluorometric assay (Thermofisher, UK), the 2100 Bioanalyser (Agilent Technology, UK), the PicoGreen measurement (Quant-it PicoGreen Kit, ThermoFisher, cat. no. P7589) and the TapeStation assay (Agilent, UK). A minimum cDNA concentration of 0.2 ng/µl was required together with a high-quality amplification profile.

### Nextera XT construction for Smart-Seq2

Samples were sent to the Wellcome Trust Centre for Human Genetics to construct the Nextera XT library (Illumina, UK). For each oocyte, three Nextera libraries were prepared. The indices to tagment the samples were added according to the Nextera DNA sample preparation kit (Illumina, Cat. no. FC-131-1096). In total, 72 libraries for 24 oocytes were pooled and run using an Illumina HighSeq 4000 instrument. The pooled cumulus cell samples were run in a separate lane. Batch effects were avoided by multiplexing. Once the samples had passed quality control, both oocytes and cumulus cells were run in the second two lanes to obtain deeper sequencing.

### Processing of reads

Quality control was carried out using FastQC and reads were aligned to the mouse genome (GRCm38[mm10]) using STAR. Samtools (Li *et al.* 2009) was used to eliminate non-uniquely mapped reads and reads that represented PCR duplicates.

### Variant calling

Reads which aligned to mtDNA were extracted and variants (where the RNA sequence differed from the consensus sequence) were called and filtered using freebayes and bcftools. Variants were only considered if the read depth at the nucleotide position was at least 100 and the minimum allele frequency was at least 1%. Multiple base substitutions and insertion/deletion events were not considered. Low quality reads were also excluded (minimum mapping quality = 20; minimum base quality = 30). The mtRNA heteroplasmy level for each variant is defined as: frequency of variant/sequencing depth at nucleotide position. We were not able to compare the incidence of the variants in oocytes and cumulus cells for a number of reasons. Firstly, the sequencing depth was greater in the cumulus cells (Supplementary Fig. 1, see section on supplementary materials given at the end of this article). Secondly the number of mitochondrial genomes in the samples of cumulus cells may be higher than that of the oocytes. Thirdly, the results may be confounded by differing transcript levels (i.e. gene expression) between oocytes and cumulus cells. Because of this, we compare only the mtRNA heteroplasmy level (i.e. percentage of the variant transcript compared to the WT transcript) and not their frequency. However, the four most common variants had sufficient read depth for robust measurement.

## Results

**Figure 1:**
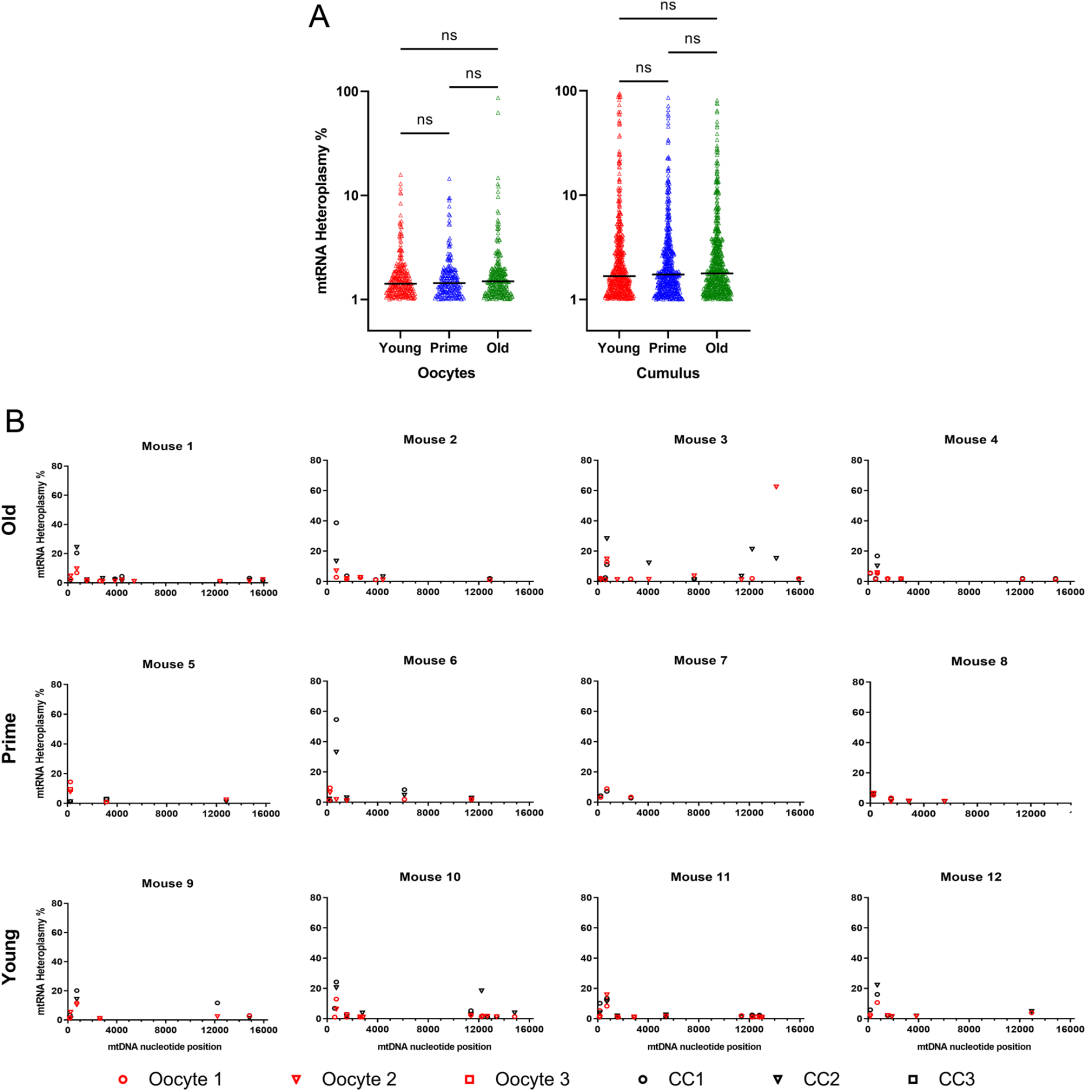
(A) All variants detected at an mtRNA heteroplasmy level >1% are shown. The majority of variants are present at levels below 10%. There are no significant differences in mtRNA heteroplasmy levels between any of the age groups in either oocytes or cumulus cells. Significance was tested using a one-way ANOVA and corrected for multiple testing using the Holm-Sidak method. (B) The mtRNA variant profile for each mouse is displayed. Only variants that were detected in more than one sample from the same mouse are shown. Each variant is shown at its nucleotide position in the mitochondrial genome. The association between the mtRNA heteroplasmy level of individual variants within the cumulus cells (CC) and oocytes can be seen. In general, the mtRNA heteroplasmy level is higher in cumulus cell samples than in oocytes from the same mouse. Due to technical error, only one oocyte-cumulus complex was collected from Mouse 7. Three oocyte-cumulus complexes were collected from Mouse 5 only.

To investigate whether there were age related differences in mtRNA heteroplasmy in the female germline, we analysed single-cell RNA-seq data from oocytes of mice aged 3 weeks, 9 weeks and 12 months. We quantified instances where the RNA sequence deviated from that of the consensus sequence (mtRNA variants). To reduce noise, we only considered mtRNA variants that were present in at least 1% of transcripts from each sample. The mtRNA variants from the eight oocytes in each age group were pooled and the results shown in Fig. 1A. The same analysis was conducted on the associated cumulus cell populations from each age group, which were analysed using bulk RNA-seq. None of the pairwise comparisons in either the oocyte or the cumulus cell samples revealed a significant difference between the age groups. This means our data do not support the hypothesis that mtRNA variants increase in abundance with age. The only transcripts where variants were present at higher levels in older mice than younger were those from the non-coding region (i.e. D-loop) (Supplementary Fig. 2). This is true in both oocytes and cumulus cells. The significance of transcripts from this region is unclear, though the data are consistent with previous studies documenting age-related accumulation of mutations in D-loop mtDNA (Arbeithuber *et al.* 2020). The distribution of the data shows that the majority of mtRNA variants in both oocytes and cumulus cells are found at low levels (<10%). Variants found at >50% (i.e. constituting a majority of the mtRNA population) were uncommon across both sample types, but were present to higher degree in cumulus cells (2/762 vs 25/1649).

**Figure 2:**
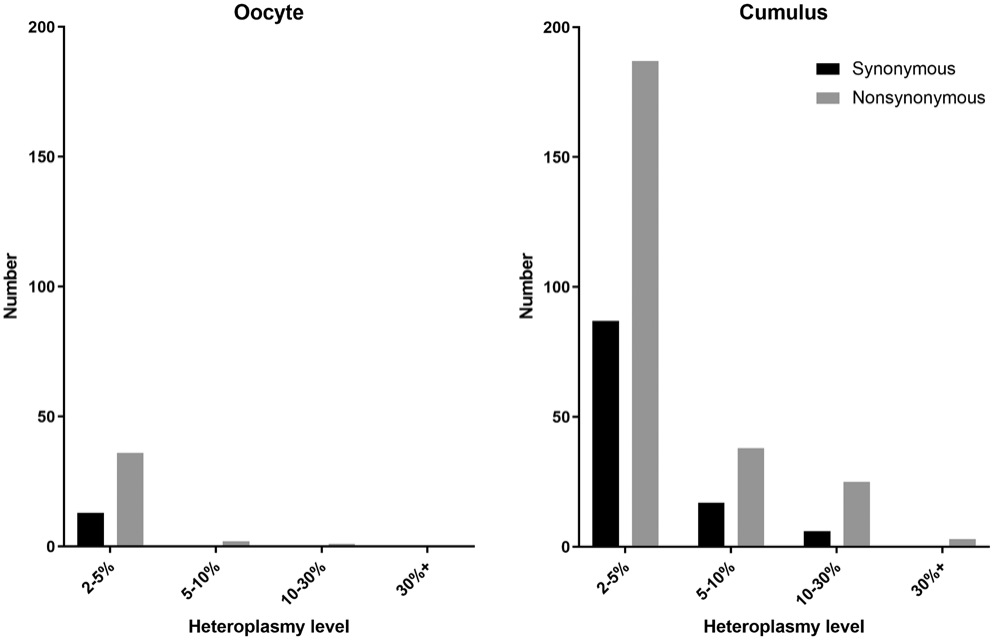
Synonymous and non-synonymous variants in oocytes and cumulus cell samples are shown, grouped by their mtRNA heteroplasmy level. Non-synonymous mtRNA variants outnumber synonymous variants in all cases.

For the rest of the analysis, we opted to focus only on mtRNA variants that were detected in more than one sample from the same mouse (i.e. present in both oocytes; present in both cumulus cell samples; or present in at least one oocyte and one cumulus cell sample). As sequencing data are notoriously noisy, selecting variants that appear in multiple samples increases the likelihood that they are true variants. These RNA variants are displayed by individual mouse in Fig. 1B. mtRNA heteroplasmies which would produce non-synonymous mutations exceeded those that would produce synonymous mutations by a factor of about two in both oocytes and cumulus cells (Fig. 2).

**Figure 3:**
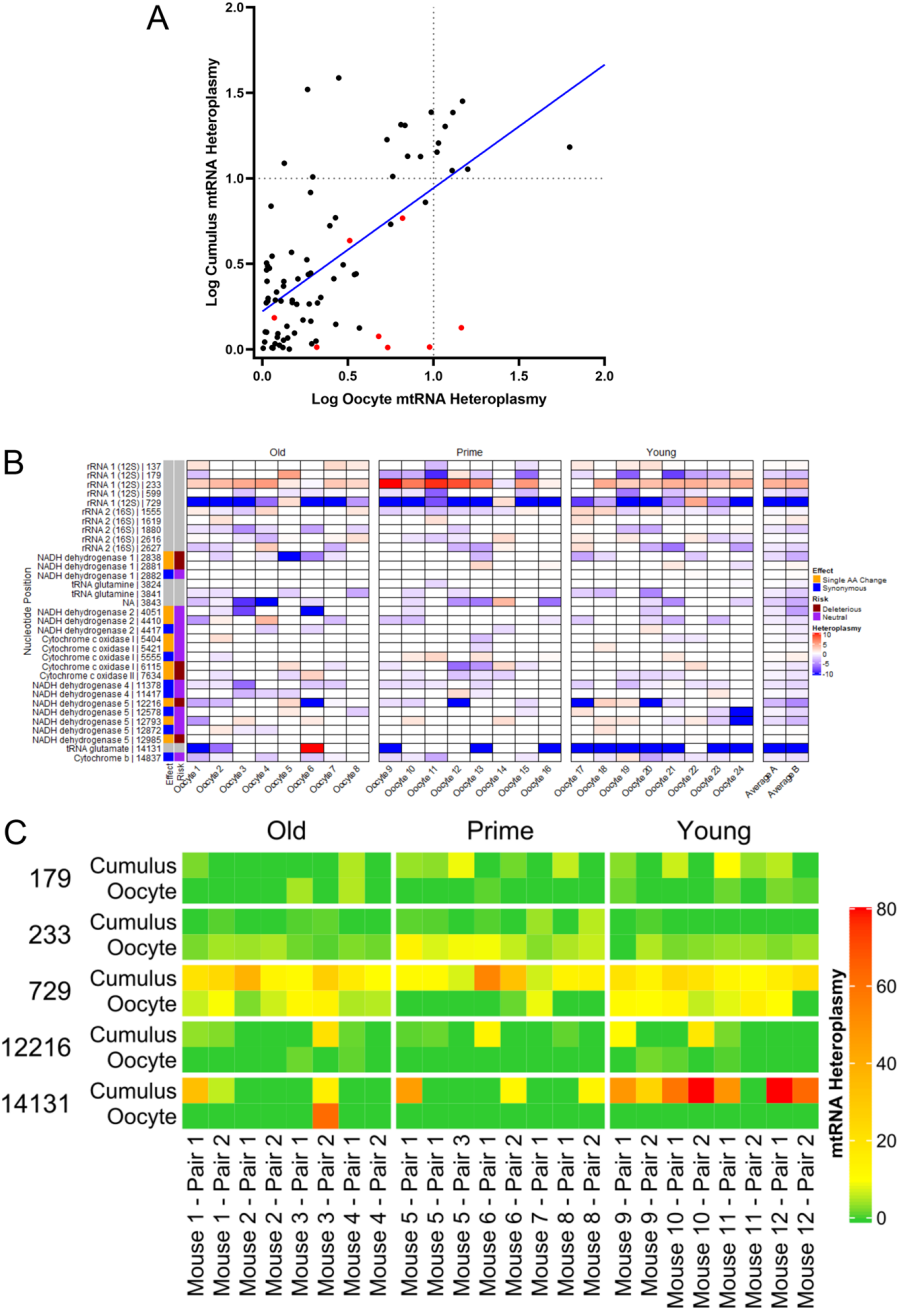
(A) There is a significant (R squared = 0.38; p value <0.0001) correlation between the mtRNA heteroplasmy level in oocytes and their associated cumulus cells (blue line). Only variants which were found in both samples from an oocyte-cumulus complex were included. The dashed lines represent a 10% mtRNA heteroplasmy level. More variant transcripts are found at >10% mtRNA heteroplasmy in cumulus cells than in oocytes. The points coloured in red represent a variant found at position 233 which lies within the *mt-Rnr1* gene. (B) Heatmap displaying the change in mtRNA heteroplasmy between cumulus cell samples and their respective oocytes. A negative (blue) value represents a higher level of mtRNA heteroplasmy in cumulus cells and a lower level in the oocyte (i.e. a reduction in heteroplasmy in the next generation). With the notable exception of the variant found at position 233, the majority of variants are found at lower levels in the oocytes. Mean average columns are displayed on the right, either including zero values (Average A) or excluding them (Average B). (C) Heatmap displaying the absolute heteroplasmy level of the five mtRNA variants which demonstrate the greatest heteroplasmy change between oocytes and their associated cumulus cells. High levels of the variant found at position 14,131 were observed predominantly in cumulus cells from young mice. Interestingly, this variant does not appear at high levels in the corresponding oocytes.

We found a significant correlation between the abundance of transcript variants in oocytes and their corresponding cumulus cells (*P* < 0.0001, Fig. 3A). Of the 75 heteroplasmic variants found in more than one sample, the majority (71/75 or 95%) appeared to be maternal in origin rather than *de novo*, as they were found in more than one sample of cumulus cells. Of these, 51/71 (72%) were detected in at least one oocyte while 20/71 (28%) were detected only in cumulus cells, or if present in oocytes then at levels of <1% heteroplasmy.

In most cases, the cumulus cells contained mtRNA variants at higher heteroplasmy level (19 variants present at >10%) compared to oocytes (9 variants present at >10%) (Fig. 3A). Accordingly, the variation between sister cumuli tended to exceed that of sister oocytes, but this was not significant between age groups (Supplementary Fig. 3). Figure 3B illustrates the change in variant load between individual cumulus cell samples and their associated oocyte. In most cases, the variant is present at lower levels in the oocytes than it is in the associated cumulus cells. The exception to this is the variant located at nucleotide position 233, which is more abundant in oocytes in 22/24 samples (*P* < 0.001). Figure 3C illustrates the five recurrent variants with the highest average mtRNA heteroplasmy level. These are located at positions 179, 233 and 729, all of which lie within the 12S rRNA encoding gene, 14131which lies within tRNAGlu, and 12,216 which is found in *mt-Nd5*. The variants at positions 729 and 14,131 show significantly lower mtRNA heteroplasmies in oocytes than cumulus cells (*P* < 0.002 and 0.005 respectively by paired sample T test), but trends in the same direction at positions 179 and 12,216 were not significant. Variant 233 is present at higher levels in oocytes than cumulus cells (*P* < 0.05). This variant is also highlighted in red in Fig. 3A. Indeed, Fig. 3A shows that sites where variant levels were higher in the oocyte than the cumulus cells were largely located in transcripts of mitochondrial rRNA genes.

## Discussion

We found no differences in mitochondrial transcript quality in oocytes from mice of different ages. In line with previous high-resolution genomic analysis of human mtRNA sequence variation (Hodgkinson *et al.* 2014, Bar-Yaacov *et al.* 2016) we did however find differences in the mitochondrial transcriptome of oocytes and cumulus cells, some of which we attribute to post-transcriptional RNA modifications. For instance, variant 233 was present at higher heteroplasmies in 22/24 oocytes compared to cumulus cells, making it more likely to be caused by post-transcriptional modification than mtDNA mutation. However, we did find enrichment of D-loop variants in older oocyte and cumulus transcripts, entirely consistent with published data on accumulation of mtDNA mutations in aging (Arbeithuber *et al.* 2020). Our findings do not reflect published mtDNA data showing accumulation of heteroplasmic variants between prepubertal and 11 month old mice (an age that corresponds with our “old” mice) (Arbeithuber *et al.* 2020), in protein coding regions, possibly because transcript differences associated with changes in the mtDNA are dwarfed by the extent of post-transcriptional modification.

Consistent with known variation in the heteroplasmic load of mtDNA in human oocytes and the corresponding cumulus cells (Boucret *et al.* 2017), most mtRNA variants were present at lower levels in oocytes than in cumulus cells. One limitation of our data is that we do not have the corresponding mtDNA sequences, so we cannot be certain which variants represent true heteroplasmy and which reflect differences in post-transcriptional modification. If our data indicate RNA modification, this would support a conclusion that post-transcriptional RNA modifications are less frequent in oocytes compared to cumulus cells, in line with their relative quiescence (Johnson *et al.* 2007). Oocyte 6 (from aged mouse 3, pair 2) was an outlier in having high mtRNA heteroplasmies at several sites, potentially reflecting its physiology. Although the external appearances of all oocytes were similar, these multiple heteroplasmies are more likely to reflect transcriptional modifications specific to this oocyte than multiple mtDNA mutations. However, because the distribution of the variants detected mirrors published data on human mtDNA variation in oocytes and cumulus cells (Boucret *et al.* 2017), we suggest that some of the observed mtRNA variation could be a consequence of underlying mtDNA heteroplasmy. Reflecting previous studies (Boucret *et al.* 2017), there was no evidence of mtDNA selection in cumulus cells because non-synonymous changes outnumbered synonymous ones (Fig. 2).

Figure 3C illustrates the five variants displaying the greatest change in abundance between oocytes and cumulus cells. Four of the five map to genes encoding RNAs and these are known to undergo a high degree of post-transcriptional modification in mitochondria (Richter *et al.* 2018). However, none of these correspond to known sites of mitochondrial rRNA modification (Hodgkinson *et al.* 2014, Bar-Yaacov *et al.* 2016) (Supplementary Fig. 4). Like most of the variants detected, 179, 729 and 14,131 are present in cumulus cells at much higher frequencies than oocytes, represented by blue shading in Fig. 3B. Variant 233 is unusual because it is increased in oocytes relative to cumulus cells (see red coloured points in Fig. 3A and red shading in Fig. 3B), but its biological significance is not yet clear.

Given the high level of post-transcriptional RNA modifications in mitochondria (Hodgkinson *et al.* 2014), it is possible that post-transcriptional modification underlies some of the observed variation. The variant located at nucleotide 14,131 maps to P9 of mitochondrial tRNA Glu, which is known to be methylated in humans (Suzuki *et al.* 2011). While this modification appears to be present in 16/24 of cumulus cell samples, and at a high mtRNA heteroplasmy level (average 23%, range 6 - 87%), it was only detected in 1/24 oocytes (Fig. 3C). In mammalian datasets, P9 methylation is required for correct folding, translational efficiency and downstream mitochondrial function of at least 11 of the mitochondrial tRNAs (Silzer *et al.* 2020). This modification may be representative of high metabolic activity in cumulus cells that is not present in oocytes. Cumulus cells are metabolically active (Boucret *et al.* 2017) and will have undergone more cell divisions than their associated oocytes since diverging from their common mother cell.

In mouse there is direct evidence that oocyte mtDNA quality changes in aging mothers (Burgstaller *et al.* 2018, Arbeithuber *et al.* 2020), but our data were not able to support this. Direct (Yang *et al.* 2020) and indirect evidence also suggest that human oocyte mtDNA quality changes over time in adult ovaries because mtDNA heteroplasmies increase in offspring with increasing maternal age at conception (Rebolledo-Jaramillo *et al.* 2014). There is direct (Floros *et al.* 2018) and indirect (Ma *et al.* 2018) evidence that the quality of mtDNA in mouse oocytes changes during prenatal development. These published data have been interpreted as evidence of mtDNA turnover (Arbeithuber *et al.* 2020) and potentially of mtDNA quality control in humans and mice (Floros *et al.* 2018). In our data, D-loop variants increased in oocytes and cumulus cells from aged mothers (Supplementary Fig. 2), but this may be an artefact as the D-loop is non-coding and hence only transcribed at low levels.

A high mtDNA mutant load can impair fertility (Yang *et al.* 2020). By supplying the energy that drives early embryogenesis, maternal mitochondria are vital components of the oocyte and accumulation of such mutations could be significant in organisms with a long lifespan. However, the variants detected in mitochondrial protein-coding transcripts were present at low levels. Hence, these are unlikely to affect mitochondrial function significantly.

In conclusion, in the first published study of mtRNA transcripts in oocytes we have shown a difference in the quality of mitochondrial RNA between oocytes and cumulus cells, potentially related to physiology, but no clear evidence that this is influenced by the age of the female. We hypothesise that this variation is partly due to differences in post-transcriptional modification which have not previously been noted in mouse oocyte RNA-seq data (Floros *et al.* 2018). We found increased variants in transcripts of the non-coding D-loop but no significant increase within protein coding regions. In the absence of mtDNA sequence data we cannot exclude the possibility of increased age-related mtDNA mutations and a larger/more sensitive study might have detected this. However, our data is consistent with less post-transcriptional modification of mitochondrial RNA in oocytes than in cumulus cells, consistent with higher metabolic activity of cumulus cells. More sophisticated methods, such as nanopore sequencing, would be needed to confirm this.

## Supplementary Material

Figure S1 - Transcript coverage in oocytes and cumulus cells. Samples taken from the same mouse are shown within a single row. The expression patterns are visibly different between oocytes and cumulus cells. Some transcripts can be seen mapping to the D-loop region (positions 15443-16299), although at relatively low levels compared to transcripts mapping to protein coding genes.

Figure S2 - Cumulative frequency of the number of variants in the D-loop present at a given mtRNA heteroplasmy level in oocytes and cumulus cells. These results suggest that variants in the non-coding region (i.e. D-loop) are present at higher levels in older mice. While this region of the genome is not represented in the major transcripts from the mitochondrial promoters, fragments mapping to the D-loop were detected in all samples. They may indicate a higher than expected rate of D-loop transcription, and possibly increased mutation of this region over time.

Figure S3 - The variation in mtRNA heteroplasmy between sister cumulus clusters (average co-efficient of variation) and sister oocytes. The mtRNA variant at 14131 was present at particularly high levels in an oocyte from Mouse 3 (old). There was no significant difference in the variation between age groups in sister cumulus cells or in sister oocytes.

Figure S4 - Known sites of post-transcriptional modification in the 12S and 16S mitochondrial rRNA genes in mouse and human. Nucleotides highlighted in yellow represent variants 179, 233 and 729 Abbreviations: pseudouridine (Psi), 5-methyluridine (m5U), 1-methyladenosine (m1A), N4-methylcytidine (m4C), 5-methylcytidine (m5C), N6,N6-dimethyladenosine (m62A), 2′-O-methylguanosine (Gm), 2′-O-methyluridine (Um).

## Declaration of interest

The authors declare that there is no conflict of interest that could be perceived as prejudicing the impartiality of the research reported.

## Funding

This work was supported by the Angus Memorial Mitochondrial Fund (ongoing generous donations from patients and their families), the Newlife Foundation for disabled children (grant number SG/14-15/11) and funding from the Royal Embassy of Saudi Arabia Cultural Bureau to Arwa Alageel. Salary support for F H was provided by the Angus Memorial Mitochondrial Fund.

## Author contribution statement

F H, J P and S W conceived the study, carried out the analysis and wrote the Ms A A carried out laboratory work. R A contributed to analysis and preparation of the manuscript. N G, E R and D G advised. S A W and J P: equal senior authors.
