# Supplementary figures and images for: No evidence for age-related differences in mitochondrial RNA quality in the female germline
Source: Reprod Fertil. 2022 Aug 24;3(3):198–206. doi: 10.1530/RAF-22-0025 (PMC9513661; doi:10.1530/RAF-22-0025)

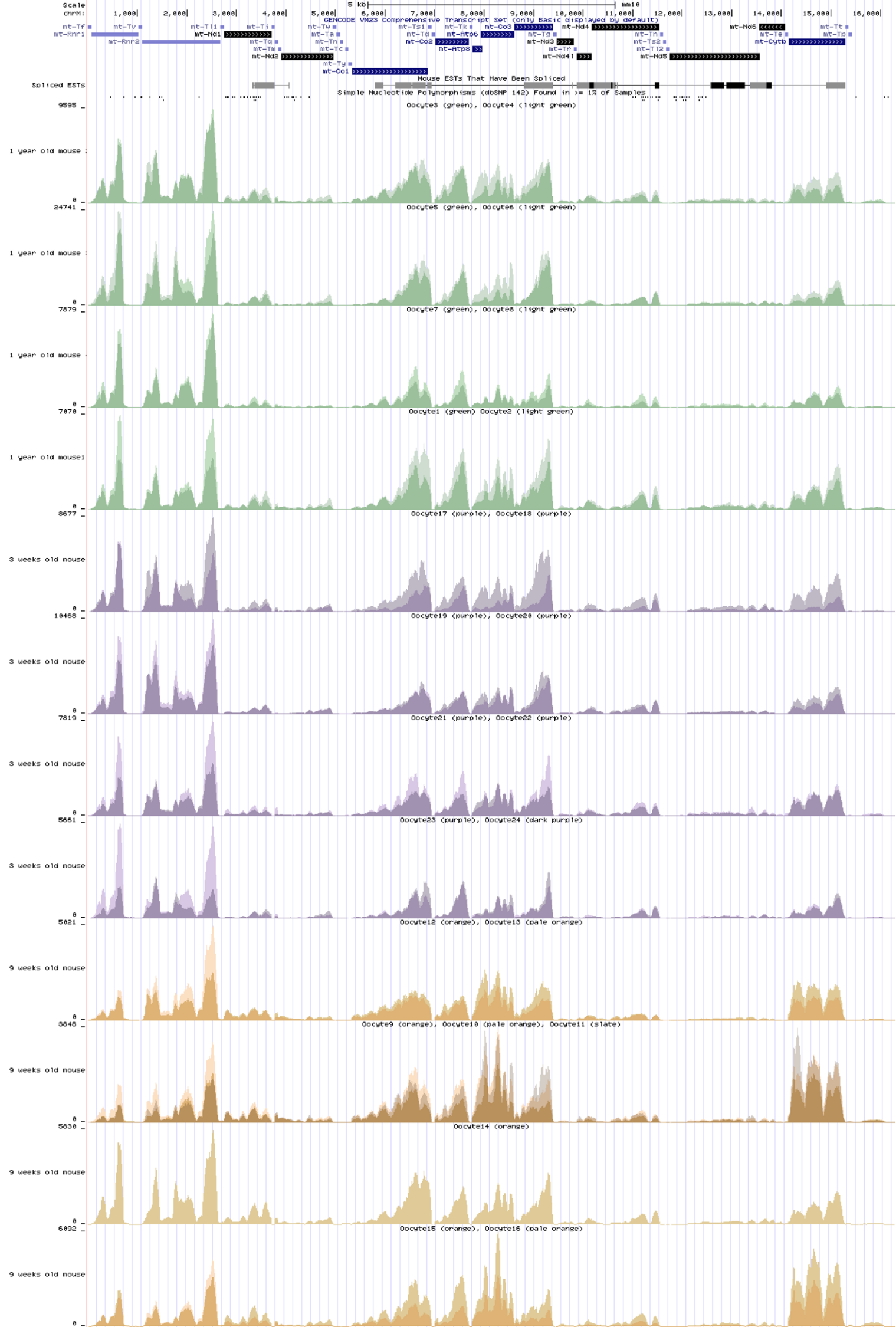

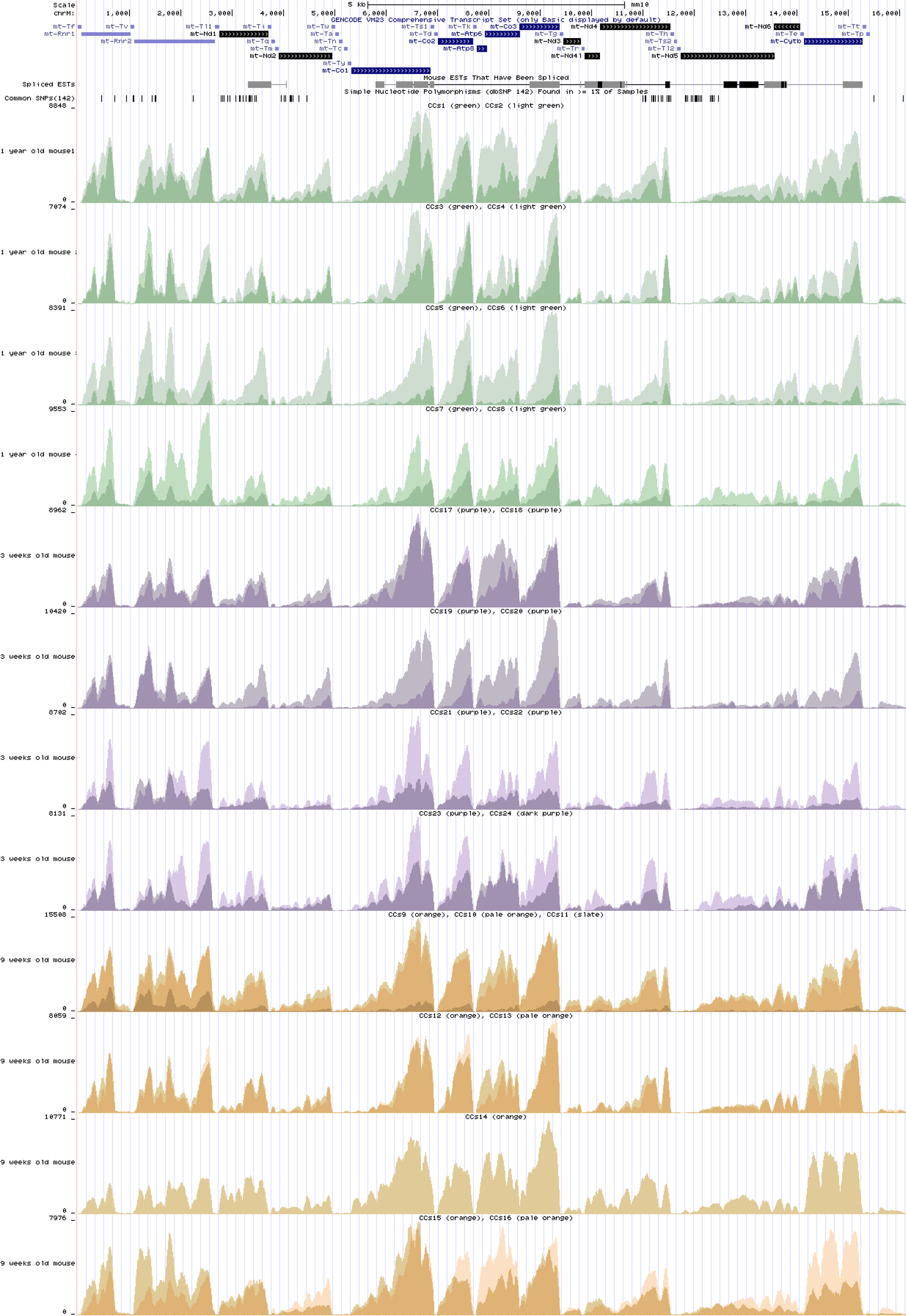

Supplement: Figure S1 - Transcript coverage in oocytes and cumulus cells. Samples taken from the same mouse are shown within a single row. The expression patterns are visibly different between oocytes and cumulus cells. Some transcripts can be seen mapping to the D-loop region (positions 15443-16299), although  [file supplementary_figure_1.pdf]

### Oocytes

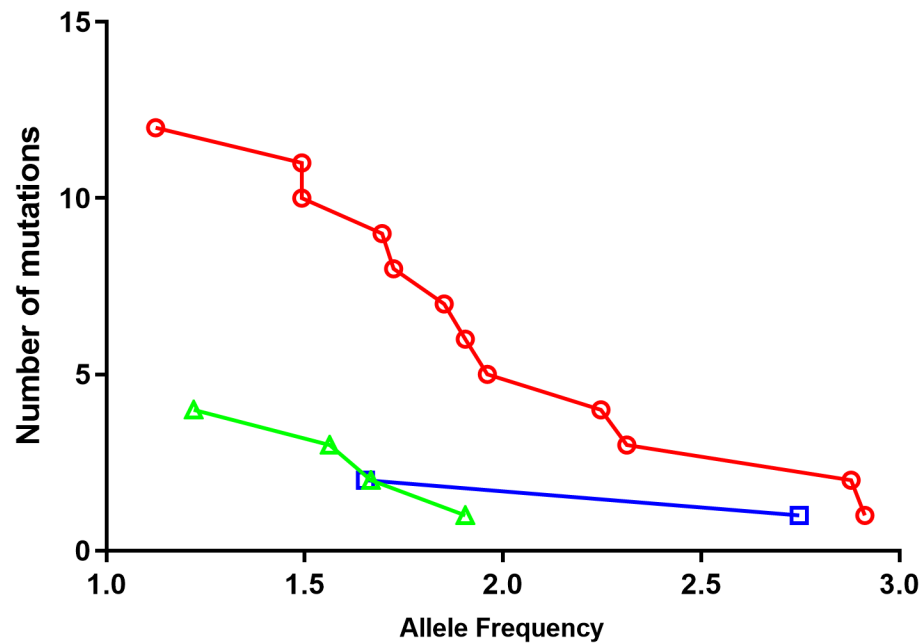

### Cumulus cells

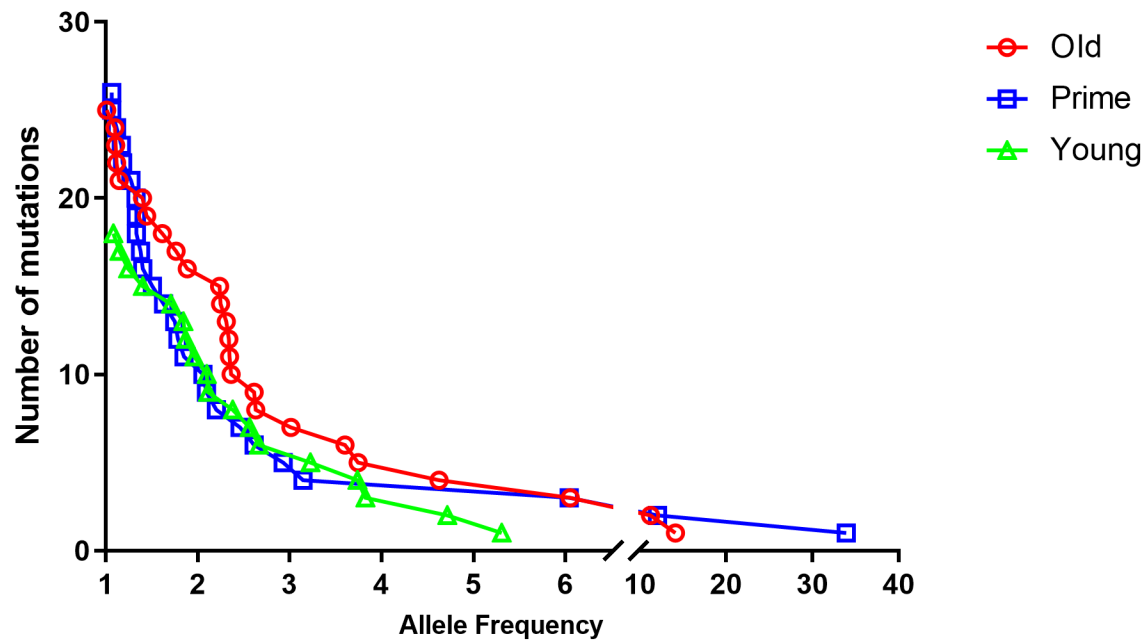

Supplement: Figure S2 - Cumulative frequency of the number of variants in the D-loop present at a given mtRNA heteroplasmy level in oocytes and cumulus cells. These results suggest that variants in the non-coding region (i.e. D-loop) are present at higher levels in older mice. While this region of the genome is [file supplementary_figure_2.pdf]

## Oocytes

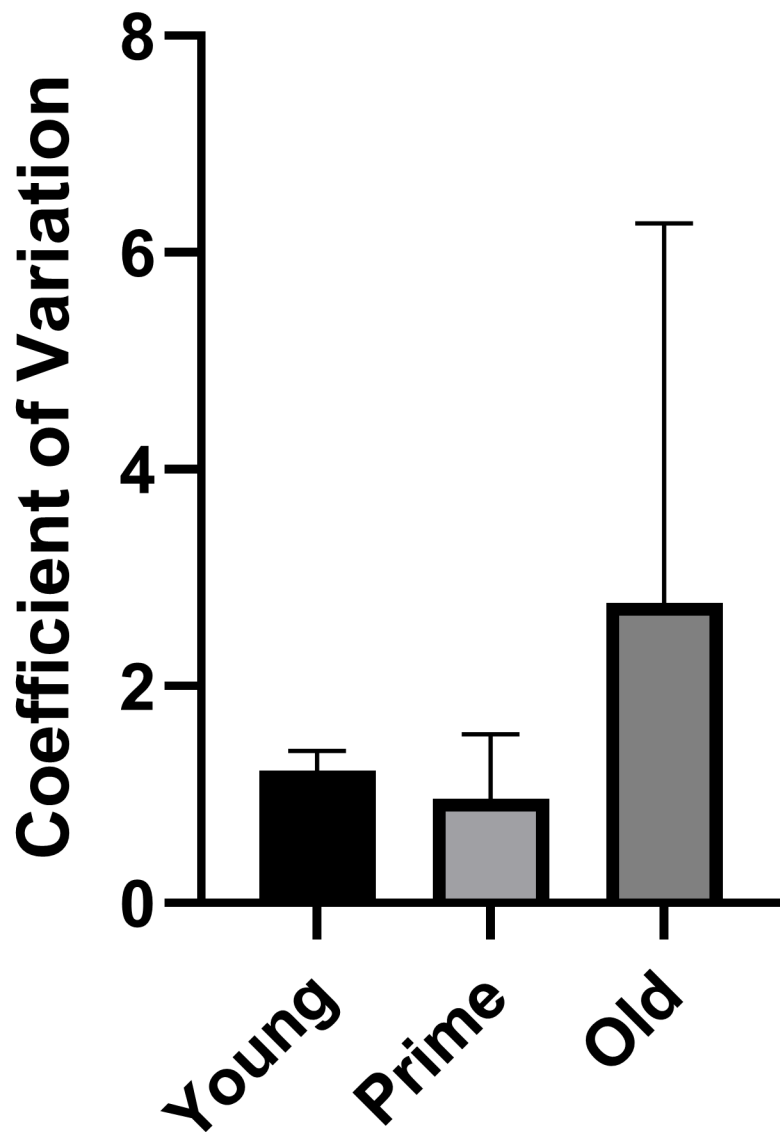

## Cumulus cells

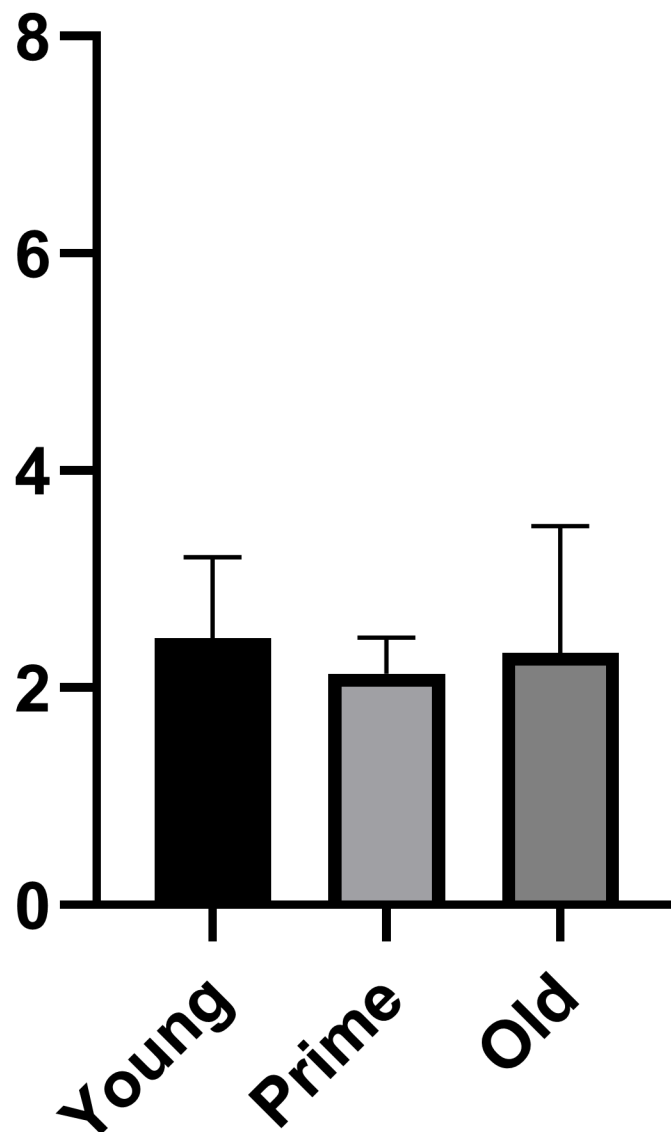

Supplement: Figure S3 - The variation in mtRNA heteroplasmy between sister cumulus clusters (average co-efficient of variation) and sister oocytes. The mtRNA variant at 14131 was present at particularly high levels in an oocyte from Mouse 3 (old). There was no significant difference in the variation between age [file supplementary_figure_3.pdf]
