# Supplementary material for: No evidence for age-related differences in mitochondrial RNA quality in the female germline
Source: Reprod Fertil. 2022 Aug 24;3(3):198–206. doi: 10.1530/RAF-22-0025 (PMC9513661; doi:10.1530/RAF-22-0025)
Supplement: Figure S4 - Known sites of post-transcriptional modification in the 12S and 16S mitochondrial rRNA genes in mouse and human. Nucleotides highlighted in yellow represent variants 179, 233 and 729 Abbreviations: pseudouridine (Psi), 5-methyluridine (m5U), 1-methyladenosine (m1A), N4-methylcytidine (m4 [file supplementary_figure_4.pdf]

>12S_Homo_sapiens
aataggtttggtcctagcctttctattagctcttagtaagattacacatgcaagcatccccgttccagtgagttcaccctctaaatcacc
acgatcaaaaggaacaagcatcaagcacgcagcaatgcagctcaaaacgcttagcctagccacacccccacgggaaacagcagtgattaa
cctttagcaataaacgaaagtttaactaagctatactaaccccagggttggtcaatttcgtgccagccaccgcggtcacacgattaaccc
aagtcaatagaagccggcgtaaagagtgttttagatcaccccctccccaataaagctaaaactcacctgagttgtaaaaaactccagttg
acacaaaatagactacgaaagtggctttaacatatctgaacacacaatagctaagacccaaactggga(**m^{5}U)** tagataccccactatgc
ttagccctaaacctcaacagttaaatcaacaaaactgctcgccagaacactacgagccacagcttaaaactcaaaggacctggcggtgct
tcatatccctctagaggagcctgttctgtaatcgataaaccccgatcaacctcaccacctcttgctcagcctatataccgccatcttcag
caaaccctgatgaaggctacaaagtaagcgcaagtacccacgtaaagacgttaggtcaaggtgtagcccatgaggtggcaagaaatgggc
tacattttctaccccagaaaactacgatagcccttatgaaacttaagggtcgaaggtggatttagcagtaaactaagagtagagtgctta
gttgaacagggccctgaagcgcgtacacaccg(**m^{4}C)** c(**m^{5}C)** gtcaccctcctcaagtatacttcaaaggacatttaactaaaacccct
acgcatttatatagaggagacaagtcgtaacatggtaagtgtactgg(**m$^6_2$A**)(**m$^6_2$A**)agtgcacttggacgaac

>12S_Mus_musculus
aaggtttggtcctggccttataattaattagaggtaaaattacacatgcaaacctccatagaccggtgtaaaatcccttaaacatttact
taaaatttaaggagagggtatcaagcacattaaaatagcttaagacaccttgcctagccacacccccacgggactcagcagtgataaata
ttaagcaataaacgaaagtttgactaagttatacctcttagggttggtaaatttcgtgccagccaccgcggtcatacgattaacccaaac
taattatcttcggcgtaaaacgtgtcaactataaataaataaatagaattaaaatccaacttatatgtgaaaattcattgttaggaccta
aactcaataacgaaagtaattctagtcatttataatacacgacagctaagacccaaactggga(**m^{5}U)** tagataccccactatgcttagc
cataaacctaaataattaaatttaacaaaactatttgccagagaactactagccatagcttaaaactcaaaggacttggcggtactttat
atccatctagaggagcctgttctataatcgataaaccccgctctacctcaccatctcttgctaattcagcctatataccgccatcttcag
caaaccctaaaaaggtattaaagtaagcaaaagaatcaaacataaaaacgttaggtcaaggtgtagccaatgaaatgggaagaaatgggc
tacattttcttataaaagaacattactataccctttatgaaactaaaggactaaggaggatttagtagtaaattaagaatagagagctta
attgaattgagcaatgaagtacgcacacaccg(**m^{4}C)** c(**m^{5}C)** gtcaccctcctcaaattaaattaaacttaacataattaatttctaga
catccgtttatgagaggagataagtcgtaacaaggtaagcatactgg(**m$^6_2$A**)(**m$^6_2$A**)agtgtgcttggaataat

>16S_Homo_sapiens
gctaaacctagccccaaacccactccaccttactaccagacaaccttagccaaaccatttacccaaataaagtataggcgatagaaattg
aaacctggcgcaatagatatagtaccgcaagggaaagatgaaaaattataaccaagcataatatagcaaggactaacccctataccttct
gcataatgaattaactagaaataactttgcaaggagagccaaagctaagacccccgaaaccagacgagctacctaagaacagctaaaaga
gcacacccgtctatgtagcaaaatagtgggaagatttataggtagaggcgacaaacctaccgagcctggtgatagctggttgtccaagat
agaatcttagttcaactttaaatttgcccacagaaccctctaaatccccttgtaaatttaactgttagtccaaagaggaacagctctttg
gacactaggaaaaaaccttgtagagagagtaaaaaatttaacacccatagtaggcctaaaagcagccaccaattaagaaagcgttcaagc
tcaacacccactacctaaaaaatcccaaacatataactgaactcctcacacccaattggaccaatctatcaccctatagaagaactaatg
ttagtataagtaacatgaaaacattctcctccgcataagcctgcgtcagattaaaacactgaactgacaattaacagcccaatatctaca
atcaaccaacaagtcattattaccctcactgtcaacccaacacaggcatgctcataaggaaaggttaaaaaaagtaaaaggaactcggca
aatcttaccccgcctgtttaccaaaaacatcacctctagcatcaccagtattagaggcaccgcctgcccagtgacacatgtttaacggcc
gcggtaccctaaccgtgcaaaggtagcataatcacttgttccttaa(**m^{1}A**)tagggacctgtatgaatggctccacgagggttcagctgt
ctcttacttttaaccagtgaaattgacctgcccgtgaagaggcgggcataacacagcaagacgagaagaccctatggagctttaatttat
taatgcaaacagtacctaacaaacccacaggtcctaaactaccaaacctgcattaaaaatttcggttg(**Gm)** ggcgacctcggagcagaa
cccaacctccgagcagtacatgctaagacttcaccagtcaaagcgaactactatactcaattgatccaataacttgaccaacggaacaag
ttaccctagggataacagcgcaatcctattctagagtccatatcaacaatagggtttacgacctcgatgttggatcaggacatcccgatg
gtgcagccgctattaaaggttcgtt(**Um) (Gm)** ttcaacgattaaagtcctacgtgatc(**Psi**)gagttcagaccggagtaatccaggtc
ggtttctatctacnttcaaattcctccctgtacgaaaggacaagagaaataaggcctacttcacaaagcgccttcccccgtaaatgatat
catctcaacttagtattatacccacacccacccaagaacagggttt

>16S_Mus_musculus
actaatcctagccctagccctacacaaatataattatactattatataaatcaaaacatttatcctactaaaagtattggagaaagaaat
tcgtacatctaggagctatagaactagtaccgcaagggaaagatgaaagactaattaaaagtaagaacaagcaaagattaaaccttgtac
cttttgcataatgaactaactagaaaacttctaactaaaagaattacagctagaaaccccgaaaccaaacgagctacctaaaaacaattt
tatgaatcaactcgtctatgtggcaaaatagtgagaagatttttaggtagaggtgaaaagcctaacgagcttggtgatagctggttaccc
aaaaaatgaatttaagttcaattttaaacttgctaaaaaaacaacaaaatcaaaaagtaagtttagattatagccaaaagagggacagct
cttctggaacggaaaaaacctttaatagtgaataattaacaaaacagcttttaaccattgtaggcctaaaagcagccaccaataaagaaa
gcgttcaagctcaacataaaatttcaattaattccataatttacaccaacttcctaaacttaaaattgggttaatctataactttataga
tgcaacactgttagtatgagtaacaagaattccaattctccaggcatacgcgtataacaactcggataaccattgttagttaatcagact
ataggcaataatcacactataaataatccacctataacttctctgttaacccaacaccggaatgcctaaaggaaagatccaaaaagataa
aaggaactcggcaaacaagaaccccgcctgtttaccaaaaacatcacctctagcattacaagtattagaggcactgcctgcccagtgact
aaagtttaacggccgcggtatcctgaccgtgcaaaggtagcataatcacttgttccttaattagggactagcatgaacggctaaacgagg
gtccaactgtctcttatctttaatcagtgaaattgacctttcagtgaagaggctgaaatataataataagacgagaagaccctatggagc
ttaaattatataacttatctatttaatttattaaacctaatggcccaaaaactatagtataagtttgaaatttcggttg**(Gm)** ggtgacc
tcggagaataaaaaatcctccgaatgattataacctagacttacaagtcaaagtaaaatcaacatatcttattgacccagatatattttg
atcaacggaccaagttaccctagggataacagcgcaatcctatttaagagttcatatcgacaattagggtttacgacctcgatgttggat
caggacatcccaatggtgtagaagctattaatggttcgtt(**Um) (Gm)** ttcaacgattaaagtcctacgtgatc**(Psi)** gagttcagacc
ggagcaatccaggtcggtttctatctatttacgatttctcccagtacgaaaggacaagagaaatagagccaccttacaaataagcgctct
caacttaatttatgaataaaatctaaataaaatatatacgtacaccctctaacctagagaaggtt
